# Genomic Patterns of Positive Selection at the Origin of Rust Fungi

**DOI:** 10.1371/journal.pone.0143959

**Published:** 2015-12-03

**Authors:** Diogo N. Silva, Sebastien Duplessis, Pedro Talhinhas, Helena Azinheira, Octávio S. Paulo, Dora Batista

**Affiliations:** 1 Centro de Investigação das Ferrugens do Cafeeiro, Instituto Superior de Agronomia, Universidade de Lisboa, Oeiras, Portugal; 2 Computational Biology and Population Genomics group, cE3c – Centre for Centre for Ecology Evolution and Environmental Changes, Faculdade de Ciências da Universidade de Lisboa, Lisbon, Portugal; 3 Departamento de Biologia e CESAM – Centro de Estudos do Ambiente e do Mar, Universidade de Aveiro, Aveiro, Portugal; 4 Institut National de la Recherche Agronomique, UMR 1136 INRA/Université de Lorraine Interactions Arbres-Microorganismes, Champenoux, France; 5 Université de Lorraine, UMR 1136, INRA/Université de Lorraine Interactions Arbres-Microorganismes, Vandoeuvre-lès-Nancy, France; 6 LEAF, Linking Landscape, Environment, Agriculture and Food, Instituto Superior de Agronomia, Universidade de Lisboa, Lisbon, Portugal; University of Lausanne, SWITZERLAND

## Abstract

Understanding the origin and evolution of pathogenicity and biotrophic life-style of rust fungi has remained a conundrum for decades. Research on the molecular mechanisms responsible for rust fungi evolution has been hampered by their biotrophic life-style until the sequencing of some rust fungi genomes. With the availability of multiple whole genomes and EST data for this group, it is now possible to employ genome-wide surveys and investigate how natural selection shaped their evolution. In this work, we employed a phylogenomics approach to search for positive selection and genes undergoing accelerated evolution at the origin of rust fungi on an assembly of single copy genes conserved across a broad range of basidiomycetes. Up to 985 genes were screened for positive selection on the phylogenetic branch leading to rusts, revealing a pervasive signal of positive selection throughout the data set with the proportion of positively selected genes ranging between 19.6–33.3%. Additionally, 30 genes were found to be under accelerated evolution at the origin of rust fungi, probably due to a mixture of positive selection and relaxation of purifying selection. Functional annotation of the positively selected genes revealed an enrichment in genes involved in the biosynthesis of secondary metabolites and several metabolism and transporter classes.

## Introduction

During the past 30 years, the Neutralist-Selectionist debate has gradually settled into a consensus that both neutral drift and selection are fundamental pillars in evolution, with positive darwinian selection playing a major role in evolutionary change [1, 2]. The action of positive selection on coding regions traditionally manifests itself by an excess of non-synonymous substitutions relatively to synonymous substitutions [3]. The search for such signatures within the genomes of organisms has been an exciting and fruitful pursuit of modern biologists, since adaptive changes in genes are ultimately responsible for evolutionary innovations that can have a significant impact on the survival and adaptation of species to their environment and generate a breath of phenotypic diversity [4]. Therefore, there is a growing interest in answering long-standing questions such as which genes were affected by positive selection, how strong was the effect, and when did it occur. Pursuing this endeavor is the first crucial step towards understanding the consequences of genetic variation on phenotypes and ultimately, its contribution to the fitness of individuals [2, 4, 5].

Plant pathogens have been obvious targets of studies aimed at finding molecular fingerprints of positive selection on avirulence genes, elicitors and effectors involved in arms-race interactions between hosts and pathogens [6]. Through this approach, several genes previously suspected to be involved in pathogenicity were also found to be under positive selection, such as the SnToxA toxin in *Pyrenophora* spp. [7], the RXLR effectors of oomycetes [8], or the AvrP4 avirulence gene of *Melampsora* spp.[9]. These studies were important to confirm and better understand how these genes affected pathogenicity, but their range was quite limited by having to rely only on previously identified candidate genes.

More recently, with the increasing availability of whole-genome sequences and sophistication of statistical methods, researchers became able to harness information from thousands of genes across multiple species and to perform genome-scans for positive selection that forego the need for *a priori* knowledge on specific genes [10]. With these advances, finding and understanding natural selection transited from hypothesis-testing to hypothesis-generating science [2]. By comparing several ortholog sequences across multiple species and employing a maximum likelihood test of dN/dS ratios, such “blind” approaches have the enormous potential of pinpointing not only genes or even amino acid residues targeted by positive selection that were not previously considered, but also the functional categories enriched for positively selected genes following a relevant evolutionary event or transition [2]. Moreover, it is now possible to uncover episodic positive selection acting on a pre-specified branch of the phylogenetic tree and, therefore, on a specific evolutionary time [11, 12]. The power and usefulness of these genome scans led to their application in a wide range of taxa to investigate a number of questions, such as differential adaptation to disease in humans and chimpanzees [13], salt adaptation in a desert poplar [14], extreme physiological adaptations in turtles [15], among other examples [16–18]. However, their application in pathogenic fungal systems has been more limited [19, 20].

In this work, a genome-wide scan for positive selection and genes undergoing accelerated evolution integrated into a phylogenomics framework was used to investigate the evolutionary origin of rust fungi (Basidiomycota, Pucciniales), namely regarding the distinctive features of their biotrophic life-style and pathogenicity. Rust fungi are a diverse and economically important group of obligate plant pathogens that cause devastating diseases on cultivated plants of almost all taxonomic families [21, 22]. Despite their importance, the obligate biotrophic life-style and inability to grow axenically has complicated the research on the molecular mechanisms responsible for the evolution of their pathogenicity. Therefore, the molecular features underlying the adaptations of obligate biotrophic associations with host plants remained largely unknown until the publication of the first two rust genome sequences, *Melampsora larici-populina* and *Puccinia graminis* f. sp. *tritici* [23]. These resources provided key insights to understand the origin and singularity of rust fungi such as: (i) expansion of lineage-specific gene families that account for a high number of predicted genes compared to other basidiomycete pathogens; (ii) absence of sucrose transporters as well as loss of some genes involved in inorganic nitrogen and sulphur uptake and assimilation; (iii) a reduced carbohydrate active enzymes repertoire, albeit some classes may be expanded; (iv) larger repertoire of small secreted proteins, most of which are also up regulated transcripts and lineage specific; and (v) transposon proliferation.

Arguably, the discovery of these genome-scale changes significantly advanced our knowledge on rust fungi, focusing on new genomic features that are unique to rust genomes [23–26]. Considerably less explored remains the role of adaptive genetic variation on genetic material shared between rust fungi and other Basidiomycetes, particularly on the phylogenetic root branch of the Puccinales. When tackling the issue of the adaptations that took place on the origin of rust fungi and their life-style, the importance of investigating variation on genes conserved across multiple species is twofold: first, it has been extensively demonstrated that even large changes of form and function can evolve by altering the sequence and functionality of conserved proteins [1, 27]; second, the genetic material of the common ancestor of rust fungi had to be shared to some extent with several contemporary populations and species, as it occurs for the common ancestors of all organisms [28]. Therefore, the first adaptive changes would have been required to occur in the shared genetic material [29]. In this work, by using sophisticated and powerful methods capable of detecting episodic positive selection and by including a more basal lineage of the Pucciniales, it was our goal to identify genes with signatures of positive selection specifically at the time when the rust fungi originated and provide insights on the strength and pervasiveness of positive selection in rust genomes.

Our specific aims were thus to: (i) detect and identify the largest number of single-copy orthologs shared among a data set of 67 Basidiomycota and Ascomyocta species with a combination of genomic and EST data; (ii) screen for episodic positive selection acting on specific amino acids and determine the magnitude of the signal for positive selection acting on the root of the rust fungi; (iii) detect genes that are significantly accelerated on the root of the rust fungi; and (iv) annotate the candidate genes and investigate if certain functional classes are enriched for positively selected genes.

## Materials and Methods

### Genomic and EST data

Genomic data was collected from 37 Basidiomycota species from public databases, as well as from 9 Ascomycota species to use as outgroups. This data set included the complete genome of three rust fungi (*Melampsora larici-populina*, *Puccinia graminis* f. sp. t*ritici* and *P*. *triticina*). Additionally, EST data from 65 Basidiomycota species was gathered from the NCBI public repository, except for *Hemileia vastatrix* [30]. In total, 753,848 EST sequences were compiled. The full list of species and their corresponding genomic sources and citations is provided in S1 Table.

### Processing of EST data

Before processing the EST data, species' sequences for which complete genome sequences were available in alternative, were removed. Twenty one species were thus excluded, resulting in an initial data set with 44 species and 259,704 sequences. ESTs were screened for vector contaminants and trimmed using SeqClean (http://seqclean.sourceforge.net/) [31] against the UniVec database (http://www.ncbi.nlm.nih.gov/VecScreen/UniVec.html), and sequences with less than 100 bp were discarded. Interspersed repeats and low complexity regions in the ESTs were then masked with RepeatMasker (A.F.A. Smit, R. Hubley & P. Green RepeatMasker at http://repeatmasker.org). To this end, the RMBlast search engine and the repeat database RepBase, which is optimized for RepeatMasker, were used. These cleaning steps resulted in a final data set of 255,156 sequences, with 51,192 Kbp trimmed and 2,080 Kbp masked.

### Ortholog search strategy

The detection of ortholog sequences in the combined genomic and EST data sets followed two sequential approaches. First, orthology relationships among 488,087 protein sequences for the 37 Basidiomycota species with complete draft genomes were assessed using the OrthoMCL software [32]. The procedure starts with all-vs-all BLASTp comparisons, for which an e-value cut-off of 1e-5 was chosen. Then, a Markov Clustering (MCL) algorithm (http://micans.org/mcl/) [33, 34] was used to create clusters of putative orthologs and co-orthologs according to an inflation parameter that controls cluster tightness. To strike a balance between cluster tightness and the probability of breaking up clusters with the same orthologs, the intermediate inflation value of 3 was used. Since the output of OrthoMCL contains recent paralogs in addition to single copy genes, clusters with more than one gene copy per species or comprising less than 9 species out of the 37 total species were removed. Furthermore, we only included single-copy genes represented by at least one species of the Pucciniales. This resulted in a final data set of 1,715 core single-copy ortholog clusters, 1,250 of which contained the three Pucciniales (S2 Table). Nine additional Ascomycota species were added for outgroup purposes. For future reference in this paper, ortholog clusters will be simply referred to as genes.

Second, the taxonomic coverage of the previously determined core single-copy genes was then expanded with EST data using the software HaMSTr [35]. By complementing only this set of genes, we attempted to substantially reduce the chance of inadvertently introduce paralogs from the EST data. Each core gene was aligned in MAFFT v7.0 [36] using the L-INS-i method and Hidden Markov Model profiles were then constructed for each alignment using *hmmbuild*, included in the HMMR3 package (http://hmmer.janelia.org) [37]. All 37 Basidiomycota species were used as representatives during the **HaMSTr** searches and the “-relaxed” option of the program was specified, in order to relax the constraint of a potential ortholog to be present in all representative species. Finally, species whose data could not complement at least 50 core genes were discarded, resulting in a final inclusion of 21 species with EST sequences. Therefore, the largest data set assembled contained genomic data from 37 Basidiomycota species, 9 Ascomycota species and EST data from 21 additional species, for a total of 67 species.

### Sequence alignment and filtering

Genes were aligned with MAFFT v7.0 using the L-INS-i method. Alignment columns with excessive missing data were filtered by a custom python script, which removed columns with a proportion of missing data above 50% in the extremities of the alignment and columns with a proportion of gaps and missing data above 75%. In order to take the alignment uncertainty into account when performing the phylogenomic reconstruction, weights were attributed to each alignment column, using the probabilistic framework implemented in ZORRO [38], for latter interpretation by the phylogenetic reconstruction software. For the detection of positive selection, the DNA sequences corresponding to each species in the protein alignments were assembled and aligned with TranslatorX [39] using the protein alignment as reference. Since the software used to detect positive selection does not incorporate the probabilistic weighting schemes from ZORRO, filtering of fast evolving and potentially misaligned alignment blocks was performed following two independent approaches: Guidance [40], using default parameters and MAFFT as the multiple sequence alignment algorithm, and Gblocks [41], within the framework of TranslatorX, using default parameters, except that columns with half gap positions were allowed (option -b5 = h).

### Data set assembly

In total, six data sets were assembled for this study with specific aims (Table 1). The first four data sets were used for phylogenetic reconstructions and contained only protein sequences. Two of these were composed of sequence data from 37 Basidiomycetes plus 9 Ascomycetes with complete genomes and they differed only on the minimum amount of species required for each gene: the *genomic46sp_sparse* data set required a minimum of 9 Basidiomycota species to be represented, while the *genomic46sp_dense* data set required at least 36 Basidiomycota species to be represented. The other two protein data sets extended the taxonomic coverage of the previous data sets with EST data from 21 additional species: the *combined7sp_sparse* extends the genes from *genomic46sp_sparse*, and *combined67sp_dense* extends the genes from *genomic46sp_dense*. The two final data sets were used for positive selection detection analyses and contained only nucleotide sequences derived from the genes in the *genomic46sp_sparse* data set but without the Ascomycota species and with the additional filter that at least one *Puccinia* spp. and *M*. *larici-populina* must be present: the *basidioPAML* data set contained sequences obtained only from complete genomes while the *basidioPAML_Hv* data set contained a subset of the sequences in *basidioPAML* for which EST data of *H*. *vastatrix* was available.

**Table 1:** Description of the datasets assembled, including the number of genes, species alignment columns and the analysis performed.

Data set	Data type	Number of genes	Number of species	Alignment columns	EST included?	Analysis
combined67sp_sparse	Protein	1715	67	775565	Yes	RAxML
combined67sp_dense	Protein	614	67	326303	Yes	RAxML
genomic46sp_sparse	Protein	1715	47	842795	No	RAxML
genomic46sp_dense	Protein	614	47	350535	No	RAxML; MCMCTree
basidioPAML	DNA	985	9–36	45–3432	No	PAML
basidioPAML_Hv	DNA	531	14–37	15–3015	Yes (*H*. *vastatrix*)	PAML

### Phylogenomic reconstruction

Maximum likelihood (ML) tree reconstruction was undertaken using RAxML v8.0.9 [42] and the PROTGAMMALG model of sequence evolution for the *genomic46sp_sparse*, *combined67sp_sparse*, *genomic46sp_dense* and *combined67sp_dense* data sets (Table 1). Node support was estimated by performing 250 non-parametric bootstrap replicates. ML searches and bootstrap replicates were performed in the CIPRES Science Gateway clusters [43].

### Detection of positive selection at the origin of the Pucciniales

Prior to the detection of positive selection, sequence alignments showing a significant amount of substitution saturation were identified and removed from further analyses using an information entropy-based index implemented in DAMBE5 [44, 45] with a p-value threshold of 0.05. In alignments containing gaps, only gap free zones were analyzed. The branch-site test of positive selection implemented in PAML v4.4 [12, 46] was then used to test for positive selection in a specific branch of the phylogenetic tree by estimating the ratio of synonymous and nonsynonymous mutations, ω. This method detects signatures of positive selection when there is an excess of non-synonymous mutations relative to synonymous mutations on any given codon and a given phylogenetic branch. Two specific branches of interest, called foreground branches, were considered in this study: (i) the one on the origin of all included Pucciniales, containing the ancient lineage of *H*. *vastatrix* (*basidioPAML_Hv*), and (ii) the one on the origin of the majority of Pucciniales with draft genomes used in this study (*basidioPAML*) (S1 Fig). By studying these two foreground branches it will be possible to assess the impact of including or excluding a basal lineage of a taxonomic group when investigating its origin, and will allow an assessment of the impact that introducing lower quality EST data may have on the results. The consensus topology of the ML tree obtained from the RAxML analyses was used in both analyses. After performing the statistical tests, sites with identical amino acid residues but divergent codons that were found to be under positive selection were discarded in order to produce a final data set containing only positively selected sites that resulted in an amino acid change.

Computations were performed with SlimCodeml [47], a faster version of Codeml optimized for the *branch-site* model, using input alignments filtered either by Guidance or Gblocks. The output of the alternative and null models for each alignment was processed with a custom pipeline, which, among other tasks, retrieved the lnL scores, calculated the log-likelihood ratios and compared them to a Chi-square distribution with one degree of freedom. To correct for multiple testing, a False Discovery Rate (FDR) correction was performed and the alternative models were considered to be significantly better than the respective null model when *q* < 0.05.

### Assessment of gene molecular rates

In parallel to the genome-wide screening of positive selection, the evolutionary rate of the genes with highest taxa representation in the *genomic46_dense* data set, was assessed for several branches of the Pucciniales by fitting a relaxed molecular clock to the data using the MCMCTree software, included in the PAML 4.7 package [46]. The phylogenetic tree resulting from the phylogenomic analyses was used as input for all alignments. Since not all species were present in all alignments, the input phylogenetic tree for each alignment was pruned according to the present taxa, with the condition that all representative species of the Pucciniales had to be included. If this condition was not verified, the alignment was discarded from the analysis. Since the approximate method of MCMCTree was mandatory for protein alignments, maximum likelihood estimates of branch lengths, the gradient vector and Hessian matrix were initially calculated using the Codeml program using the WAG+Γ_4_ substitution model. Then, these calculations were used by MCMCTree to estimate divergence times and substitution rates on the tree topology. The calibration point on the root of the input phylogenetic tree was fixed to an arbitrary value of 5 (i.e., the prior was a uniform distribution with [5,5] bounds). Each analysis of an individual alignment was run twice to ensure convergence of the results and the mean of the gamma prior on the overall substitution rate was verified in each case to ensure that it was of the same magnitude as the estimated substitution rate.

To assess whether a certain gene is evolving at a faster rate in the ancestral branch of all rust fungi, the Grubb's test for outliers was used. This statistical test is able to detect outlier values in a univariate data set. Therefore, genes were considered to be evolving at an atypical substitution rate at the root branch of the Pucciniales when the test statistic, Grubbs G, for that value was below a rejection cut-off of 0.05.

### Functional annotation

To gain insight on the functions of both the putative positively selected and genes under accelerated evolution, a functional annotation was performed using the euKaryotic Orthologous Group (KOG) terminology, according to the eggNOG 4.0 [48] database and using its BLAST-based online search tool (http://eggnog.embl.de). Additionally, a Gene Ontology enrichment analysis was performed to determine if any category was overrepresented for genes under positive selection and faster evolutionary rate. Statistically significant enrichment was tested against a reference of all genes analysed using the Fisher's exact test and a p-value for the independence of rows and columns in a 2x2 contingency table was computed. Significance was considered for *p* < 0.10.

To collect a more detailed information on those genes, sequence homology searches were performed against several databases: the NCBI non-redundant (nr) nucleotide and protein databases (www.ncbi.nlm.nih.gov), the genome sequences of *Melampsora larici-populina*, *Puccinia graminis* f. sp. *tritici* and *Uromyces fabae* [23, 49] and the Pathogen-Host Interaction (PHI-base v3.2) reference database ([50]; www.phi-base.org). BLAST searches were conducted with an e-value cut-off of e^-5^, and only the best hit was considered.

## Results

### Assembly of phylogenomic data sets

Six data sets were assembled for this study with different purposes and information concerning the number of genes, species and alignment columns is provided in Table 1. The two data sets pairs *genomic46sp_sparse*/*combined67sp_sparse* and *genomic46sp_dense*/*combined67sp_dense*, were used for phylogenomic analyses, the data set *genomic46sp_dense* was used for the evolutionary rate analysis and the data sets *basidioPAML* and *basidioPAML_Hv* were used for the positive selection detection analyses. Information about missing data and average gene length for each data set is provided in S2 Table.

For the phylogenomic reconstruction the two pairs of data sets composed of protein sequences with and without translated EST data were used to assess evolutionary relationships and the potential impact of EST sequences on the phylogeny: (i) *genomic46sp_sparse*/*combined67sp_sparse* data sets contained 1,715 genes with at least 9 Basidiomycota species represented; and (ii) *genomic46sp_dense*/*combined67sp_dense* data sets comprised 614 genes with a minimum of 36 Basidiomycota species represented. The data sets that contained EST data presented an expected high level of missing amino acid data (*combined67sp_sparse*: mean = 42.0%, standard deviation = 36.2; *combined67sp_dense*: mean = 34.49%, standard deviation = 38.2) in great part due to the fragmented and limited distribution of EST sequences across the core genes. In contrast, the average missing amino acid data of the corresponding genome-only pairs was relatively low according to phylogenomic standards (*genomic46sp_sparse*: mean = 27.4%, standard deviation = 19.0; *genomic46sp_dense*: mean = 13%, standard deviation = 10.7). For the assessment of evolutionary rates, only genes from the *genomic46sp_dense* data set were used, since the higher sequence error rate of EST sequences could artificially increase estimates of substitution rate and produce misleading results.

Concerning the two data sets composed of nucleotide sequences, the *basidioPAML* data set was composed of 985 genes from the *genomic46sp_dense* data set, with at least one *Puccinia* sp. and *M*. *larici-populina* represented and after the exclusion of 21 genes with significant substitution saturation. In an effort to include an ancient lineage of the Pucciniales, the previous data set was complemented with EST data of *H*. *vastatrix*. The *basidioPAML_Hv* data set contained 531 out of the 985 genes for which sequence data of *H*. *vastatrix* was available and after the exclusion of 11 saturated genes. The average proportion of missing genes per species was low in both data sets (*basidioPAML*: mean = 5.0%, standard deviation = 6.0; *basidioPAML_Hv*: mean = 3.9%, standard deviation = 4.8) as well as the proportion of missing nucleotides in the alignments (*basidioPAML*: mean = 11.8%, standard deviation = 5.6; *basidioPAML_Hv*: mean = 4.9%, standard deviation = 8.0). The average sequence length was also similar between data sets (*basidioPAML*: mean = 736, standard deviation = 12; basidioPAML_*Hv*: mean = 825, standard deviation = 64). Even though the average proportion of missing data was smaller in the *basidioPAML_Hv* data set, the sequence data of *H*. *vastatrix* presented a substantial amount of missing data (45.8%), mainly due to the presence of gaps in the alignments, that also led to a substantial reduction in the average alignment length in this data set (364 bp ± 290, compared to the 712 bp ± 520 of the *basidioPAML* data set).

### Evolutionary history of the Pucciniales

**Fig 1:**
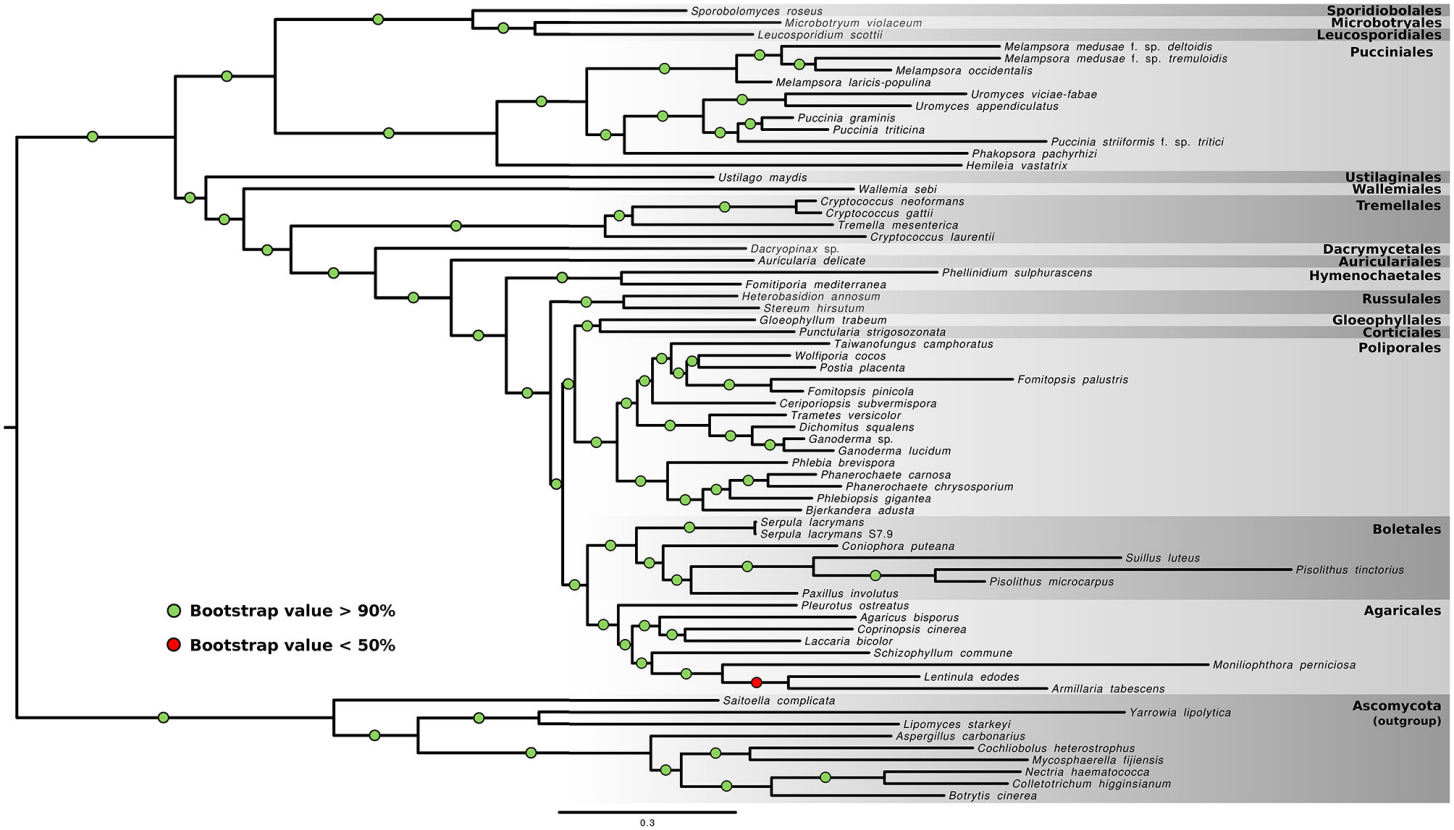
Basidiomycota phylogenetic tree. Maximum likelihood tree estimated illustrating the evolutionary relationships among 67 Basidiomycota and Ascomycota species. Species that contain only EST data are followed by an asterisk. Names on the right of the figure correspond to the taxonomic order of the respective highlighted taxa. Within the Pucciniales, the three sub-orders defined in [51] are also presented.

In a phylogenomic attempt to resolve the phylogenetic backbone of the Pucciniales, Maximum Likelihood (ML) phylogenetic reconstructions were performed. The ML trees obtained among the four data sets were congruent and presented a fully resolved phylogeny with maximum bootstrap support for all branches, except for the position of *Armillaria tabescens* and *Lentinula edodes* in the Agaricales (Fig 1). The inclusion of EST data did not have an effect on the general topology of the tree when compared to the trees derived from complete genome data only, despite the substantial increase in missing data. In the Pucciniales, the three previously proposed main sub-orders [51] were recovered with strong support as well as with the same branching order, with *H*. *vastatrix* diverging early within the rust fungi taxonomic order.

### Episodic positive selection at the origin of the rust fungi

Detection of episodic positive selection was undertaken using two data sets aimed at studying the evolutionary origin of the Pucciniales but targeting two different foreground branches. While the *basidioPAML* data set targets the foreground branch leading to the most recent common ancestor of the *Melampsora* and *Puccinia* genera, the *basidioPAML_Hv* data set, which includes EST data from *H*. *vastatrix*, targets the foreground branch representing the most ancestral split among the Pucciniales. Based on the Guidance filtered alignments, signatures of positive selection were uncovered in 328 genes (33.3% of the data set), after the FDR correction, in the *basidioPAML* data set. In the *basidioPAML_Hv* data set 104 (19.6%) genes were found to be under positive selection for the same FDR threshold. When the same *branch-site* analysis was performed on Gblocks filtered alignments, signatures of positive selection were found in 216 genes (21.9%) for the *basidioPAML* data set and 100 genes (18.8%) for the *basidioPAML_Hv*. Considering both analyses, 177 (82%) positively selected genes were mutually detected in the *BasidioPAML* data set and 78 (78%) in the *BasidioPAML_Hv* data set. Since Guidance was shown to outperform Gblocks as an alignment filtering tool for positive selection detection analyses, particularly due to its ability to recover false negatives [52], only results from Guidance filtered alignments will be further explored. Notwithstanding, the complete results of the Gblocks filtered alignments are provided in S1 Text.

Since the branch-sites model detects episodic selection acting on the amino acid level, information on the number and profile of the selected amino acids can also be obtained to provide deeper insights. In the *basidioPAML* data set, 2,067 sites were found to be under positive selection with a Posterior Probability (PP) above 0.95 across 274 genes (27.8%), while in the *basidioPAML_Hv* data set, 289 selected sites were also detected across 72 genes (13.6%).

To further explore the profile of the selected amino acid sites on both data sets, two main site classes were established to assess their potential adaptive role: (i) *Unique*, sites containing a single variant exclusive to the Pucciniales (strict) or rarely found outside the Pucciniales (relaxed); and (ii) *Diversifying*, sites containing multiple variants exclusive to the Pucciniales (strict) or rarely found outside the Pucciniales (relaxed). Therefore, each site class comprises two sub-classes referring to sites sorted in a strict or relaxed fashion.

**Fig 2:**
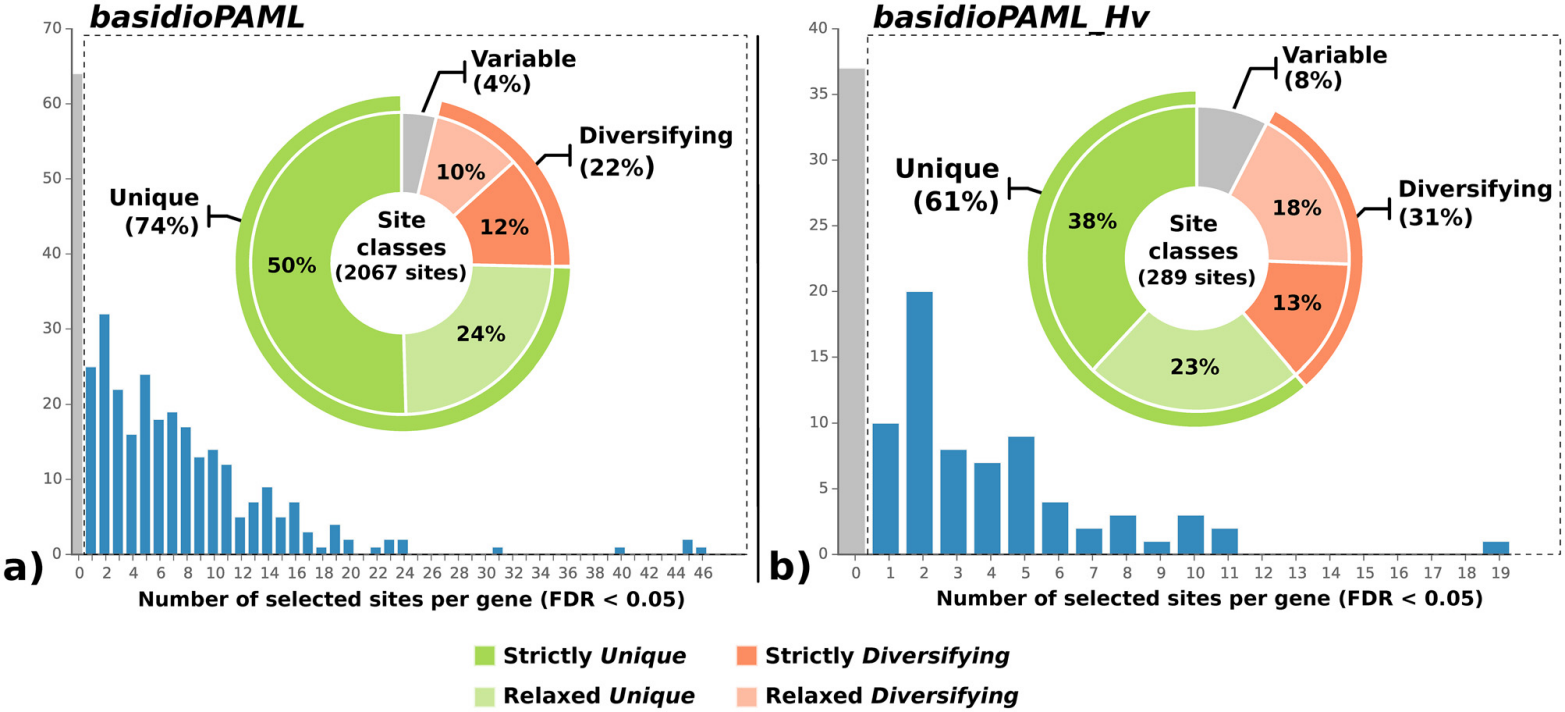
Distribution of positively selected sites. Distribution of the number of positively selected amino acid sites after correction of p-values with a false discovery method for the data set containing three rust genomes (*basidioPAML*) (a) and the data set containing the same rust genomes in addition to EST data from *Hemileia vastatrix* (*basidioPAML_Hv*) (b). Embedded in each histogram is a doughnut chart with the distribution of the positively selected sites across the two main site class pairs defined in this study for the *basidioPAML* data set (a) and *basidioPAML_Hv* data set (b). *Unique* sites represent amino acids exclusive and identical in all rust species and *Diversifying* sites represent amino acids exclusive but variable in rust species. The site classes are colour coded with the corresponding legend on the bottom of the figure.

**Fig 3:**
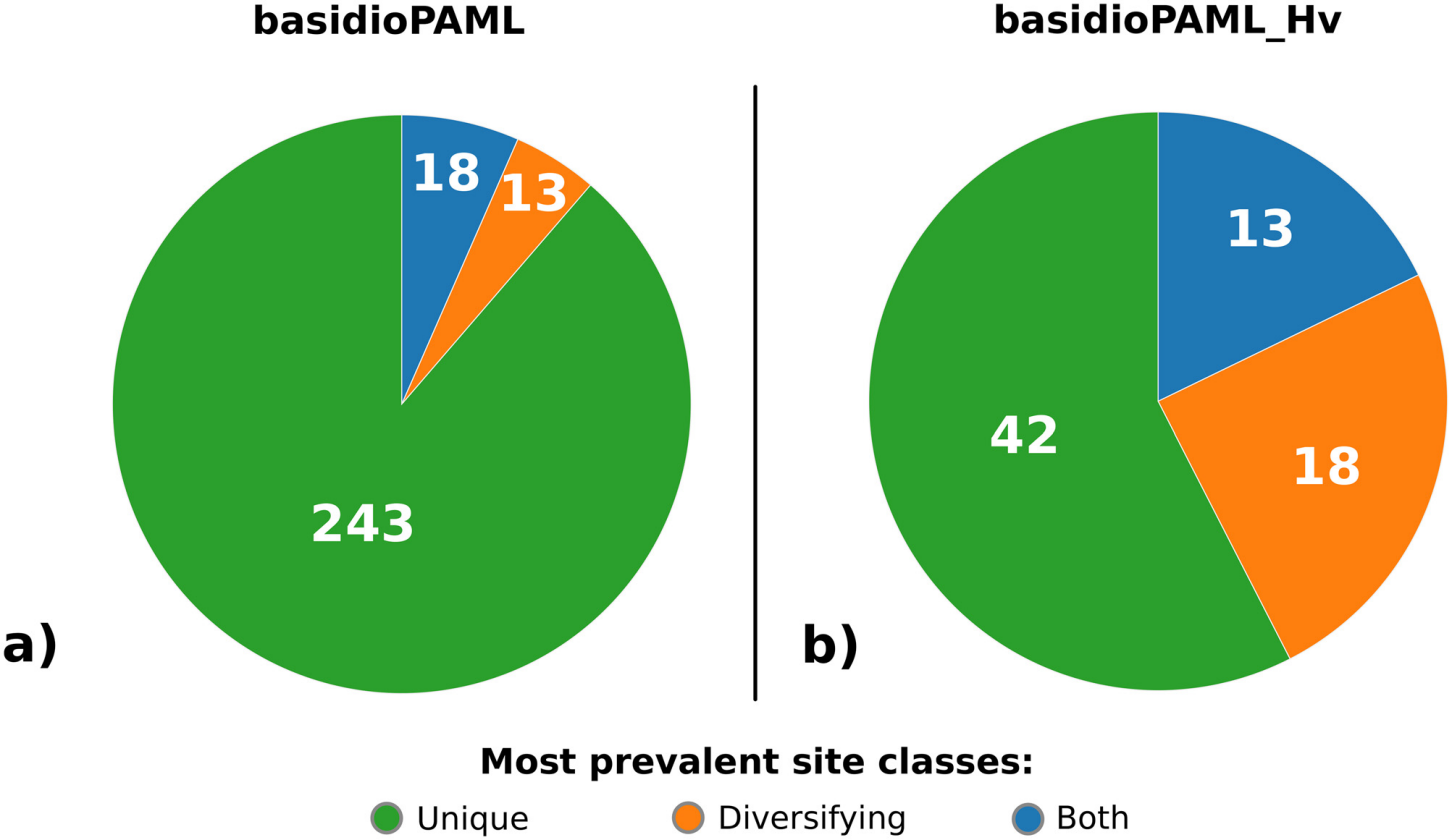
Prevalence of positively selected site classes. Pie charts with the distribution of the most prevalent site classes across each positively selected gene for the data set containing only the three rust genomes (*basidioPAML*) and the data set containing the same rust genomes in addition to EST data from *Hemileia vastatrix* (*basidioPAML_Hv*). Site classes are colour coded according to the legend in the right.

The distribution of the number of selected sites per gene and the proportion of sites in each class is summarized in Fig 2. In both data sets, the majority of the positively selected sites could be assigned to either *Unique* or *Diversifying* classes [*basidioPAML*: 1,991 (96%); *basidioPAML_Hv*: 267 (92%)]. In the *basidioPAML* data set, most sites were assigned to the *Unique* class (1538 sites, 74%, across 259 genes) even though a non-negligible proportion of *Diversifying* sites were uncovered (453 sites, 22%, across 156 genes). Regarding the *basidioPAML_Hv* data set, there was an increase in the proportion of *Diversifying* sites (90 sites, 31%, across 43 genes) but the majority of the selected sites were still placed in the *Unique* class (177 sites, 61%, across 62 genes). Only a small proportion of sites (4% for *basidioPAML* and 8% for *basidioPAML_Hv*) could not be assigned to neither class. Since the same gene can have positively selected sites from both classes, a distribution of the most prevalent site class per gene is presented in Fig 3. While *Unique* sites account for the majority of selected sites in both data sets, the advantage is less pronounced in the *basidioPAML_Hv* data set.

Comparing both data sets 75 genes had signatures of positive selection in both data sets, while 253 genes were exclusive from the *basidioPAML* data set and 29 were exclusive from the *basidioPAML_Hv* data set.

A database containing information about the PAML tests for each alignment, including the position and number of selected sites is provided in S3 Table.

### Estimation of relative evolutionary rates

Substitution rates were estimated using a Bayesian framework for all branches in 614 gene trees represented in most Basidiomycota species in this study. Two trees were excluded from this analysis as they did not include all three Pucciniales species. The mean relative substitution rate of the Pucciniales' root branch was found to be similar to the average substitution rate over all branches and gene alignments (root branch mean = 0.40; all branches mean = 0.43), but the standard deviation found in the root branch was much higher than in the overall substitution rate in all branches (root branch standard deviation = 4.49; all branches standard deviation 0.59). No genes were identified as being evolving at a significantly slower rate in the root branch of the Pucciniales, but 30 genes (5%) were found to be substantially accelerated, compared to the rates of the remaining branches of the corresponding gene. From these genes under accelerated evolution, only 13 were also found to be under positive selection using the branch-site model and these genes were not necessarily the fastest. For example, in the top ten of the fastest evolving genes, only three had signatures of positive selection.

### Functional annotation and enrichment

Overall, 357 unique genes (75 shared by both data sets, 29 exclusive from the *basidioPAML_Hv* data set and 253 exclusive from the *basidioPAML* data set) were detected as being under positive selection in either foreground branches. Functional annotation of these 357 genes was obtained using the KOG terminology (S4 and S5 Tables). Over 82% (292) of the positively selected genes were classified into 21 specific KOG categories, while 10% (36) had no specific KOG category assigned [“Function unknown” (13) or “General function prediction only” (23)], and 8% (29) had no hits. Considering the proportion of genes under positive selection against the respective reference of all orthologs analysed, statistical analysis of the under or overrepresentation of the targeted genes for the *basidioPAML* revealed a relative enrichment (over 1.5 fold change) in genes annotated into “Secondary metabolites biosynthesis, transport and catabolism”, “Amino acid transport and metabolism”, “Coenzyme transport and metabolism” and “Nuclear structure”, and an impoverishment (less than 0.67 fold) in genes annotated into “Chromatin structure and dynamics”, “Signal transduction mechanisms” and “Cytoskeleton” (S3 Table). However, the Fisher's exact test revealed that only the “Amino acid transport and metabolism” functional class was significantly enriched in positively selected genes.

**Fig 4:**
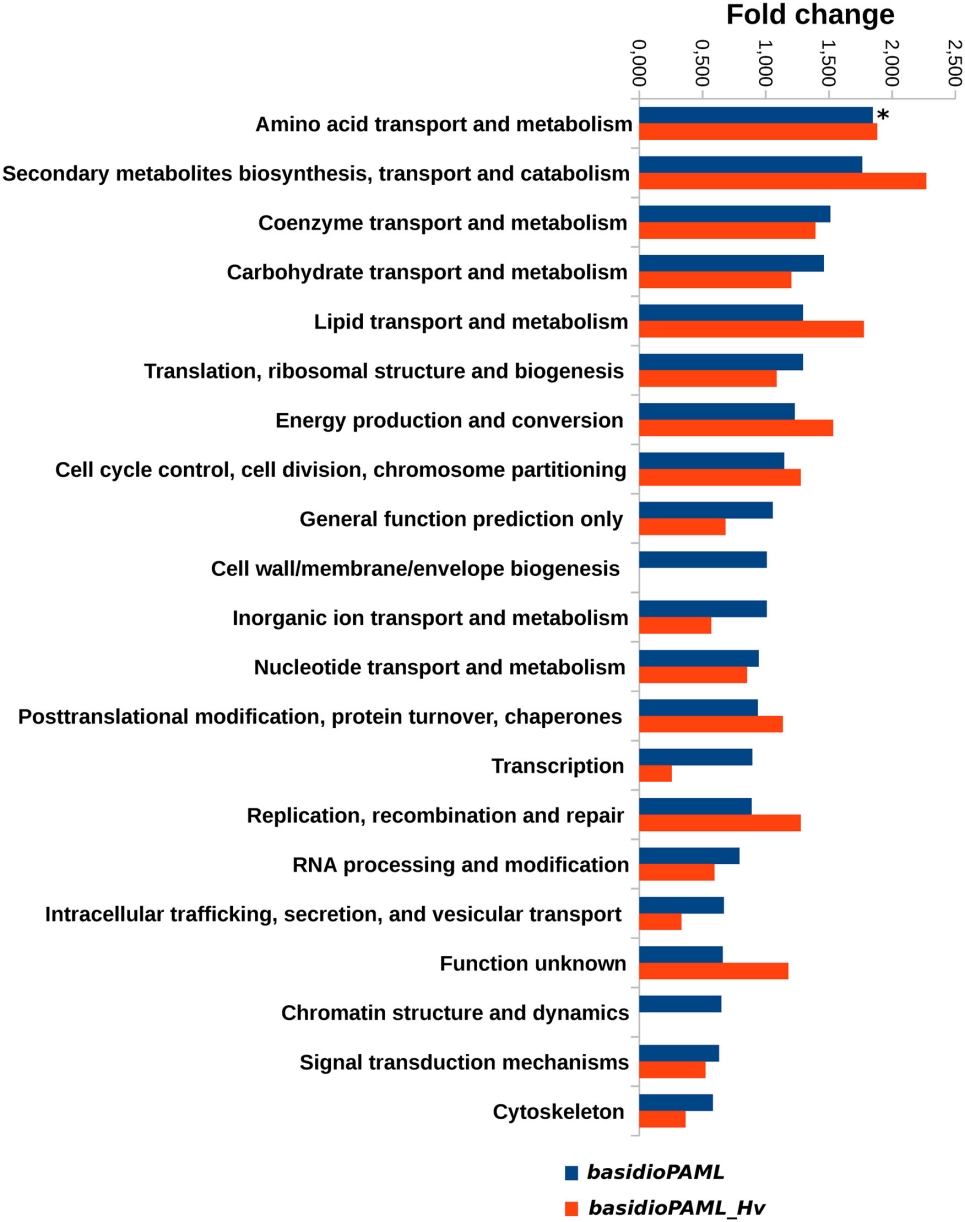
Enrichment of functional categories among positively selected genes. Bar chart with the fold change comparing the proportion of genes under positive selection and without positive selection in the y-axis, and the several KOG functional categories in the x-axis. For each functional category, fold change values are presented for the *basidioPAML* and *basidioPAML_Hv* data sets, according to the legend in the top right corner of the figure. Bars with an asterisk (“*”) represent statistically significant results for *p* < 0.1.

For the *basidioPAML_Hv* dataset, KOG annotation relative enrichment (over 1.5 fold change) was found in genes as assigned to “Secondary metabolites biosynthesis, transport and catabolism”, “Energy production and conversion”, “Lipid transport and metabolism” and “Amino acid transport and metabolism”, as well as an impoverishment (less than 0.67 fold) in genes annotated as “Inorganic ion transport and metabolism”, “Transcription”, “RNA processing and modification”, “Intracellular trafficking, secretion, and vesicular transport”, “Signal transduction mechanisms” and “Cytoskeleton”, (Fig 4). However, no functional classes were significantly over or under represented in this data set according to Fisher's exact test.

To allow a direct comparison between the *basidioPAML* and *basidioPAML_Hv* data sets, we performed the same enrichment analysis for the *basidioPAML* data set with the 531 non-saturated shared genes among data sets as reference in order to account for differences within the same universe of reference genes. Similarly to the respective whole set of orthologous, genes annotated as “Secondary metabolites biosynthesis, transport and catabolism” and “Amino acid transport and metabolism”were enriched as well as genes from the “Carbohydrate transport and metabolism” class. Genes annotated as “Replication, recombination and repair” and “Cytoskeleton” were also found to be impoverished, in addition to “Intracellular trafficking, secretion and versicular transport”. Fisher's exact test reported a statistically significant enrichment of the “Amino acid transport and metabolism” functional class as for the complete *basidioPAML* data set, but further revealed a significant enrichment of ‘Carbohydrate transport and metabolism’ and impoverishment of ‘Signal transduction mechanisms’.

Among the studied 985 genes, 249 blasted with genes involved in pathogenicity at PHI-base. Out of the 328 positively selected genes in the *basidioPAML*, 93 presented homology with entries in the PHI-base as well as 35 out of the 104 genes under positive selection in the *basidioPAML_Hv*. In both data sets, the Fisher's exact test revealed that positively selected genes were enriched for PHI-base assigned genes putatively involved in pathogenicity, with p-values of < 0.0001 for both data sets.

## Discussion

In this work, the role of adaptive genetic variation underlying the origin of the rust fungi (Basidiomycota, Pucciniales) was investigated by developing a phylogenomic framework that assembled and analyzed hundreds of single copy ortholog genes across a broad range of Basidiomycota. A crucial prerequisite for this undertaking is the establishment of a robust phylogeny across the studied Basidiomycota species, particularly of the most basal branches within the Pucciniales. Molecular systematics of the rust fungi has been considerably less explored than for other groups of fungi, but previous studies using a multi-loci approach proposed a division of the order into three sub-orders, Uredinineae, Melampsorineae and Mikronegeriineae. [51, 53, 54]. On par with Ebersberger et al. [55], our study represents one of the first attempts to use phylogenomics to resolve relationships within the Pucciniales with congruent results, but our data set furthermore includes representatives from all three sub-orders. As previously reported, *H*. *vastatrix* was placed at the base of the Puccinales in the Mikronegeriineae sub-order, confirming the importance of its inclusion when studying the origin of the rust fungi order [51].

### Genome-wide scan for positive selection

After establishing a backbone phylogeny for the studied Basidiomycota, up to 985 non-saturated genes were screened for signatures of positive selection using the *branch-site* model. This method revealed to be particularly well suited for our investigation, as it is capable of detecting episodes of positive selection specifically on the root branch of the Pucciniales. An analysis pipeline was then constructed to perform a genome scan on two different but related data sets. The *basidioPAML* data set included 985 genes solely retrieved from complete draft genomes but did not include *H*. *vastatrix*. From those 985 genes, the *basidioPAML_Hv* data set consisted in a sub-sample of 531 genes that could be complemented with EST data from *H*. *vastatrix*. Therefore, even though our pipeline analyzed the root branch of the Pucciniales in each data set, they represent distinct evolutionary periods, with the *basidioPAML_Hv* data set providing the most ancient branch but also significantly less data than the *basidioPAML* data set. Both data sets presented an impressively high proportion of positively selected genes, suggesting an unexpectedly pervasive role of positive selection shaping the origin of rust fungi: 328 genes (33.3%) showed signatures of positive selection in *basidioPAML* as compared to 104 genes (19.6%) in *basidioPAML_Hv*. Even when the same alignments were filtered with Gblocks, which is known for its aggressive removal of alignment columns and consequent reduction in power to detected sitewise positive selection, the number of positively selected genes remained high [216 genes (21.9%) for *basidioPAML* and 100 genes (18.8%) for *basidioPAML_Hv*]. Studies that employ genome scans to search for positive selection using the *branch-site* model are numerous but they generally present lower proportions of genes under positive selection. Examples from cetaceans, turtles and fig wasps report proportions of positively selected genes ranging between 4.8–6.2% [15, 16, 56]. Only in a study of humans and chimpanzees was this proportion raised to 16.6%, but this accounts for results across several branches of the phylogenetic tree [13]. In fungi, the only examples of genome scans aiming at detecting positive selection presented values ranging between 3.2–9% in *Microbotryum* spp. [19, 20], but in these studies the *sites* model was used to detect positive selection, which requires the signal to be averaged over all branches [12, 57]. The discrepancy between our results and those found in literature may lie in the fact that most of these studies were focused on comparisons between closely related taxa and/or on terminal branches. Indeed, it is well documented that the power of selection detection methods decays quite rapidly when studying closely related species or short branches that have not accumulated sufficient substitutions [1, 58]. In our analyses, positive selection is being searched on a set of non-saturated but substantially divergent sequences and in both data sets the targeted branch is fairly long, which increases the likelihood of finding positive selection. On the other hand, an increase in the number of false positives is also an unavoidable consequence of aligning difficult sets of highly divergent sequences [52], and their occurrence in our data set cannot be ruled out. However, this issue was addressed to the extent that it was possible through the application of several rigorous quality checks and data filters on all alignments. These included the removal of saturated alignments, removal of sites with excessive missing data and alignment filtering using Guidance and Gblocks. Given the high number of statistical tests, all original *p*-values of the branch-site tests were also corrected using a False Discovery Rate method. Therefore, we suggest that the higher proportion of positively selected genes in the root of the Pucciniales may be mainly explained by both a biologically relevant role of positive selection on the origin of this taxonomic group as well as an increase in selection detection power in our data sets.

To further investigate how positive selection might have acted during the early evolution of rust fungi, we took advantage of the ability of the branch-site model to detect selection on specific amino acids on the branch of interest. For the *basidioPAML* data set, 2,067 sites were detected to be under positive selection across 274 genes (27.8%), as compared to the 289 sites across 72 genes (13.6%) on the *basidioPAML_Hv* data set. While the lower proportion of positively selected genes and sites in the latter data set may be attributed to biologically relevant differences that arise from studying different foreground branches, this may also be a reflection of differences in the composition of the data sets, such as the average gene length reduction in the data set containing *H*. *vastatrix*, as regions with potentially selected sites may not be present in the sequences of this species.

We then explored the profile of the selected amino acids by sorting them among two main site classes: *Unique* and *Diversifying*. By establishing these classes, the aim was to shed light on the adaptive role of the selected sites and how they may have contributed to the evolution of rust fungi. *Unique* and *Diversifying* positively selected sites are potential prime candidates for adaptive drivers underlying the initial divergence and adaptation of rust fungi, but their distinction may be key to distinguish between adaptations that are important for all rust species and remain unchanged throughout their evolution and adaptations that later diversified in different terminal branches of the Pucciniales. Most of the positively selected sites showed a *Unique* class pattern in both data sets, though its proportion decreases in the *basidioPAML_Hv* data set, with the inclusion of the ancient *H*. *vastatrix* branch, which is also reflected on the prevalence of each site class in the positively selected genes. This result is expected because the *basidioPAML_Hv* data set includes more divergent species, but it also stresses the importance and impact of including an ancient lineage when studying the evolutionary history of a taxonomic group. In either case, this seems to reveal that adaptive changes that occur at the root of the Pucciniales are more likely to remain conserved across all rust species possibly in order to conserve a newly acquired adaptive trait. On the other hand, some positively selected sites were subject of further modification since the origin of rust fungi and may be responsible for species or genus specific alterations, although it cannot be concluded whether such modification was due to a relaxation of selective pressure or continuous action of positive selection. Nevertheless, our results show that 95% and 91% of the positively selected genes in *basidioPAML* and *basidioPAML_Hv* data sets, respectively, contain both *Unique* and *Diversifying* sites, suggesting that natural selection may shape the same gene in different ways with potentially different outcomes during the evolution of a species group. This new layer of information provided by modern methods for the detection of positive selection at the macro-evolutionary scale may allow researchers to move beyond the simple identification of genes under positive selection and grant them the power to detect the specific sites targeted by natural selection and how their variants evolve henceforth. Indeed, this information should be valuable for future studies investigating the functional aspects of the positively selected genes detected in this study for rust fungi, since they will provide the most likely sites responsible for functional change.

### Assessing the evolutionary rate on the origin of Pucciniales

To further investigate the evolutionary rate on the basal branch of the Pucciniales, we assessed the substitution rate on that specific branch across the 612 genes with the highest taxa representation in our data set, including all three Pucciniales species with complete genomes. *Hemileia vastatrix* was not included because the fragmented and overall lower quality nature of the EST data could bias estimates of substitution rates and produce misleading results. On average, the molecular rate of the root branch of the Pucciniales seems to be evolving on par with the average molecular rate of all other branches in the tree, but a significant heterogeneity in the substitution rates was also present, with a strong bias towards fast evolving genes since all outlier genes were evolving more rapidly. This stems from the fact that a single point substitution rate per gene is being compared to an average over all substitution rates per gene that tends to homogenize a sample. Thirty genes (5%) were found to be significantly accelerated but only 13 were also found to be under positive selection. While it is easy to conceive that a gene found to be under positive selection may not be under an accelerated substitution rate, particularly if positive selection acts upon a limited number of sites, the explanation for the absence of positive selection on genes under accelerated evolution at the amino acid level is less straightforward. However, two explanations seem plausible. One can be attributed to the assembly of the data sets themselves. Since tests for positive selection are more sensitive to misaligned and ambiguous alignment columns, these were filtered with Guidance for the SlimCodeml analyses but not for the MCMCTree analyses. Therefore, alignment regions where selection could actually be occurring may be visible in the evolutionary rates analysis, but absent in the branch-site test. By repeating the branch-site test for data sets without Guidance filtering the number of overlapping genes between analyses raised to 18 (data not shown), suggesting that the signature of positive selection could be indeed present in the complete alignments of some genes under accelerated evolution. The second possible explanation can be attributed to a relaxation of purifying selection on these genes, rather than the action of positive selection. In a case of relaxed purifying selection, the rate of nonsynonymous mutations does not overly exceed the rate of synonymous mutations and therefore, there is no signature of positive selection. Indeed, individual inspection of branch-site test results for the non-overlapping genes revealed that the rate of synonymous substitution was high relatively to non-synonymous substitutions, supporting the scenario of relaxed purifying selection. In such case, the relaxation of purifying selection would allow for a greater accumulation of both synonymous and non-synonymous substitutions, which would produce the observed results. Such genes could be under a relaxation of purifying selection due to temporary loss of function [59], subfunctionalization associated with gene duplication [60], or as precursors of phenotypic plasticity [61]. Even though these genes lack the intriguing signature of positive selection, they should warrant further investigation as to why purifying selection was suddenly relaxed on genes that are conserved across a broad range of Basidiomycota species.

### Functional enrichment analyses

The impact of studying different foreground branches that span different time periods between the *basidioPAML* and *basidioPAML_Hv* data sets was further explored by investigating the enrichment of functional classes for the positively selected genes in each data set. Given the substantial overlap of positively selected genes between data sets (75 genes), the enriched functional classes were also found to be similar. The metabolic related classes of “Amino acid transport and metabolism” and “Secondary metabolites biosynthesis, transport and catabolism” were found to be the most enriched for positively selected genes in both data sets, with the first mentioned class showing statistical significance in the *basidioPAML* data set. When comparing directly these data sets at the level of the 531 shared genes, we found that the “Carbohydrate transport and metabolism” class was also significantly enriched for the *basidioPAML* data set. Therefore, enrichment of functional classes related to the metabolism of amino acids, carbohydrates and secondary metabolites seems to be a common trend across both data sets and is likely to reflect a key process of adaptation that rusts in general faced during their early evolution. We further note that the positively selected genes assigned to these enriched classes were very similar between data sets. Nevertheless, differences were also found for functional classes such as “Lipid transport and metabolism” and “Energy production and conversion”, both of which presented higher fold changes in the *basidioPAML_Hv* data set. These differences may be a reflection of natural selection targeting slightly different functional classes during the early evolution of all Pucciniales.

In such a focused group of plant pathogens it is not surprising to find that positive selection may have favoured genes directly related with pathogenicity. In fact, the positively selected genes in both data sets were enriched for genes with PHI-base assignment, which have been previously demonstrated to be involved in plant-pathogen interactions and to have known roles in the infection process of several fungal species. Even though the pathogenicity related genes present in PHI-base have been mostly characterized in different fungal species, which makes extrapolations less straightforward, this provides a potential link between our positively selected genes and their contribution to the pathogenic process. For instance, genes shown to produce a mutant phenotype of loss of pathogenicity were found to be under positive selection in both evolutionary times, such as BasidioOnly3480 gene, encoding a UDP-glucose dehydrogenase (EC:1.1.1.22), ortholog to *ugd1* gene required for the pathogenicity of *Cryptococcus neoformans* [62]; only in the most ancestral branch, such as gene BasidioOnly3181, encoding a 3-isopropylmalate dehydrogenase, ortholog to *leu2* gene that reduces virulence in *Saccharomyces cerevisiae* [63]; and only in the most recent foreground branch studied, such as gene BasidioOnly3451, encoding a mitochondrial homoaconitate hydratase, ortholog to the *Aspergillus fumigatus* gene *lys*F required for pathogenicity [64]. Moreover, among the seven selected genes comprised in the enriched category “Secondary metabolites biosynthesis, transport and catabolism” in both data sets, five are referenced as associated with loss of pathogenicity or reduced virulence in *Magnaporthe grisea*, *Stagonospora nodorum* and *Colletotrichum lagenarium* [65, 66]. These include gene BasidioOnly2693, encoding an ATP-binding cassette transporter, ortholog to the multidrug resistance efflux pump *ABC*3 gene of *Magnaporthe grisea*, which is required for host penetration and for survival during oxidative stress mounted by the host through the efflux of toxic compounds [66], or BasidioOnly3214, ortholog to *THR1* reductase gene of *Colletotrichum lagenarium*, essential for the appressorium melanization process, which is a requirement for successful host penetration [65]. Interestingly, rust fungi and other biotrophs have suffered a loss of secondary metabolism genes, possibly due to the reduction in the need of degrading plant cell wall biomass and availability of amino acids from the living host [67]. Moreover, since these genes may also be capable of triggering an effective host defense their removal may reduce the opportunity to elicit a rejection from the host [67]. It is thus possible that the action of positive selection on these genes is another way for the pathogen to overcome the host defenses by introducing variation that avoids recognition by the host.

Positively selected genes were also found to be enriched in genes involved in “Amino acid transport and metabolism”, encoding enzymes with crucial roles which catalyze important steps or regulate several biosynthetic pathways. Most of the genes assigned to this functional category showed a signature of positive selection only in the foreground branch leading to the most recent common ancestor of the *Melampsora* and *Puccinia* genera (*basidioPAML*). These include genes codifying for key step enzymes, such as a putative ATP phosphoribosyltransferase (BasidioOnly3456), catalyzing the first step and controlling the rate of histidine biosynthesis (EC 2.4.2.17).

Interestingly, genes encoding two of the three key enzymes governing polyamine metabolism are represented in this list (BasidioOnly3343 predicted ornithine decarboxylase (ODC) (E.C.4.1.1.17) and BasidioOnly3535 predicted S-adenosylmethionine decarboxylase (SAMDC) (E.C.4.1.1.50)) [68]. Moreover, a gene (BasidioOnly3294) annotated as a predicted arginase, which converts arginine into ornithine, the substrate of ODC, was also found to be under positive selection but only in the most ancestral foreground branch. The two decarboxylases are the rate-limiting enzymes of polyamine biosynthesis playing a central role on the fine tune regulation mechanism controlling intracellular polyamines pools. It has been demonstrated that during host-fungus interaction, polyamine metabolism suffers striking changes in response to infection [68]. Polyamines are essential for growth and have been implicated in the regulation of both cell proliferation and differentiation processes, such as dimorphism, spore germination, appressorium formation and conidiation [69]. By modulating development and differentiation, in some way or another, polyamines regulate the virulence of animal and plant fungal pathogens. Bailey et al. [70] have shown that the *ODC* gene is essential in *Septoria nodorum* to obtain polyamines from the host plant for normal growth during infection. Additionally, *Ustilago maydis* mutants affected in the *SAMDC* gene were shown to be completely avirulent to maize [71]. An enrichment in positively selected genes involved in amino acid metabolism is consistent with a high primary metabolism activity observed in the invading rust fungi [23], since acquisition of nutrients for the development of haustoria within the host plant is crucial to the success of rust pathogen biotrophic interactions.

Positively selected genes belonging to the “Carbohydrate transportation and metabolism” class were also found to have an impact on pathogenicity of other fungi. The BasidioOnly3559 gene, ortholog to *tps1*, encodes for a synthase subunit of the trehalose-6-P synthase/phosphatase complex which synthesizes the storage carbohydrate trehalose. It was shown in *Magnaporthe oryzae*, that *tps1* has regulatory functions that control the expression of virulence associated genes and plays a pivotal role in the establishment of plant disease [72]. The BasidioOnly4049 gene encodes for a serine/threonine protein phosphatase, ortholog to *Saccharomyces cerevisiae sit4* gene, which is important for hyphal growth and virulence in *S*. *cerevisiae* through the regulation of cell wall biogenesis, osmosensing and protein translation [73]. The *ugd1* gene (BasidioOnly3480 predicted UDP-glucose dehydrogenase) is required for the metabolism of UDP-glucuronic acid, which in turn is essential for the capsule formation and pathogenicity of *Cryptococcus neformans* [74]. In general, genes involved in “Carbohydrate transportation and metabolism” are important since very early stages of fungal pathogenicity, including the mobilization of nutrients from spores for fungal development, but also for melanin biosysthesis in appressoria formation, lytic activity and camouflage. For instance, the activity of chitin deacetylases, with convert fungal chitin to chitosan, avoiding recognition by host chitinases, enables fungal camouflage and contributes to pathogenicity [30].

Overall, these results reveal the usefulness and predictive power of detecting positive selection over conserved genes that apparently have a significant role in the evolution of rusts in general and may have been involved in their origin. Other genomic features and patterns unique to the rust fungi and associated with their origin, such as gene duplication/loss patterns, were also recently identified [26]. In their study, Pendleton et al. [26] revealed that the origin of the rust fungi was associated with the loss of a substantial number of genes (1217) and a relatively small number of duplications (248). This is also consistent with our observation that the rust species included in our work shared less orthologs with other Basidiomycetes than any other Basidiomycota species between each other. However, despite the considerable gene family losses and contractions at the origin of the group, the three individual rust species included in Pendleton et al. [26] (*Cronartium quercuum* f. sp. *fusiforme*, *Melampsora lirici-populina* and *Puccinia graminis* f. sp. *tritici*) exhibited disproportionally high amounts of species-specific gene duplications. Considering these results along with the pervasive signal of positive selection found in our study, this suggests that the transition of the rust fungi to obligate biotrophy required major changes in both the composition of gene families and in the nucleotide sequences of conserved genes, followed by rapid species specific changes that would enable adaptation to their hosts. In the future, it will be interesting to investigate if similar patterns underlie the origin of other phylogenetically unrelated obligate biotrophs. Our framework also provided a useful and comprehensive hypothesis generator data for future studies focusing on the functional impact of the adaptive changes found in these genes. Indeed, further detailed investigation of the genes targeted by natural selection on the origin of rust fungi, particularly those related to metabolism, may yield new insights on the precursors of the main features of rust fungi, and these insights can be compared and verified in other biotrophic taxa. Additionally, the site classes for the positively selected amino acids defined in our study provide an additional and interesting layer of information that will require attention when investigating exactly how changes in the nucleotide sequences translate into specific adaptations and impact the fitness of individuals. It will be interesting to see if the proportions of *Unique* and *Diversifying* site classes are similar across a wide range of taxa. Despite the large number of genes, our study has detected positive selection on a single main branch of the Pucciniales. As more rust genomes get sequenced, it will become possible to investigate the action of positive selection not only on single branches but, more excitingly, on all branches since their origin up to the most terminal taxa in order to see how natural selection is shaping genes along the entire evolutionary history of rust fungi.

## Supporting Information

S1 Fig Schematic representation of the different foreground branches being considered in the *basidioPAML* and *basidioPAML_Hv* data sets. (EPS)

S1 Table Overview of genomic and EST data. Detailed information on the genomic and EST data used on the present study for each species with the taxonomic rank, total number and length of protein sequences, link to genome database and corresponding citation.
(XLS)

S2 Table Summary statistics of alignments for all data sets. Summary statistics about missing data information and average gene length for each of the six data sets used in this study.
(XLS)

S3 Table PAML results. Detailed table with the results of the branch-site test for each gene contained in the *basidioPAML* and *basidioPAML_Hv* data sets.
(XLS)

S4 Table Overview of functional annotation, selection and evolutionary rates tests. Table containing information regarding the results of the functional annotation, positive selection and evolutionary rate tests for each gene contained in the *basidioPAML* and *basidioPAML_Hv* sets.
(XLS)

S5 Table Enrichment analyses overview. (XLS)

S1 Text Description of the “Episodic positive selection at the origin of the rust fungi” results for the Gblocks filtered alignments. (DOCX)
