# Supplementary material for: Genomic Patterns of Positive Selection at the Origin of Rust Fungi
Source: PLoS One. 2015 Dec 3;10(12):e0143959. doi: 10.1371/journal.pone.0143959 (PMC4669144; doi:10.1371/journal.pone.0143959)
Supplement: S1 Text — (DOCX) [file pone.0143959.s007.docx]

**Supplementary text 1**: The following text describes the results for the “Episodic positive selection at the origin of the rust fungi” and “Functional annotation and enrichment” subsections, concerning the Gblocks filtered alignments.

**Episodic positive selection on at origin of the rust fungi**

Detection of episodic positive selection was undertaken using two data sets aimed at studying the evolutionary origin of the Pucciniales but targeting two different foreground branches. While the *basidioPAML* data set targets the foreground branch leading to the most recent common ancestor of the *Melampsora* and *Puccinia* genera, the *basidioPAML_Hv* data set, which includes EST data from *Hemileia vastatrix*, targets the foreground branch representing the most ancestral split among the Pucciniales. For the former data set, significant substitution saturation was found on 21 genes, which were removed from the subsequent analyses. Significant signatures of positive selection were uncovered in 216 genes (21.9% of the data set), after the FDR correction. In the later data set, 11 genes were removed due to substitution saturation. From the remaining 531 genes, 100 (18.8%) genes were found to be under positive selection for the same FDR threshold. Both data sets presented similar proportions of positively selected genes but the branch-sites model detects episodic selection acting on the amino acid level and thus information on the number and profile of the selected amino acids can also be obtained to provide deeper insights. In the *basidioPAML* data set, 1152 sites were found to be under positive selection with a Posterior Probability (PP) above 0.95 across 186 genes (18.8%), while in the *basidioPAML_Hv* data set, 241 selected sites were also detected across 68 genes (12.8%).

To further explore the profile of the selected amino acid sites on both data sets, two main site classes were established to assess their potential adaptive role: (i) *Unique*, sites containing a single variant exclusive to the Pucciniales (strict) or rarely found outside the Pucciniales (relaxed); and (ii) *Diversifying*, sites containing multiple variants exclusive to the Pucciniales (strict) or rarely found outside the Pucciniales (relaxed). Therefore, each site class comprises two sub-classes referring to sites sorted in a strict or relaxed fashion.

The distribution of the number of selected sites per gene and the proportion of sites in each class is summarized in Fig. 2. In the *basidioPAML* data set, most sites were assigned to the *Unique* class (863 sites, 75%, across 169 genes) even though a non-negligible proportion of *Diversifying* sites were uncovered (240 sites, 21%, across 94 genes). Regarding the *basidioPAML_Hv* data set, there was an increase in the proportion of *Diversifying* sites (87 sites, 35%, across 40 genes) but the majority of the selected sites were still placed in the *Unique* class (144 sites, 60%, across 52 genes). Since the same gene can have positively selected sites from both classes, a distribution of the most prevalent site class per gene is presented in Fig. 3. While *Unique* sites account for the majority of selected sites in both data sets, the advantage is less pronounced in the *basidioPAML_Hv* data set.

Comparing both data sets, the overlapping of positively selected genes is relatively low, since only 49 genes had signatures of positive selection in both data sets. On the other hand, 166 genes were exclusive from the *basidioPAML* data set and 51 were exclusive from the *basidioPAML_Hv* data set.

A database containing information about the PAML tests for each alignment, including the position and number of selected sites is provided in Supplementary table 3.

**Functional annotation and enrichment**

Functional annotation of the 268 genes detected as under positive selection in both foreground branches was obtained using the KOG terminology (Supplementary data 4 and 5). Over 79% (212) of the positively selected genes were classified into 21 specific KOG categories, while 12% (31) had no specific KOG category assigned [“Function unknown” (13) or “General function prediction only” (18)], and 9% (25) had no hits. Considering the two sets of annotated selected genes separately, the comparison of the proportions of genes in the different KOG classes against the respective reference of all orthologous genes analysed for each dataset, revealed that the functional classes with a higher proportion of genes under positive selection are “Translation, ribosomal structure and biogenesis” for the *basidioPAML* dataset and “Posttranslational modification, protein turnover, chaperones” for the *basidioPAML_Hv* dataset. Statistical analysis of the under or overrepresentation of the targeted genes for the *basidioPAML* revealed however a relative enrichment (over 1.5 folds) in genes annotated into “Secondary metabolites biosynthesis, transport and catabolism”, “Amino acid transport and metabolism”, “Cell wall/membrane/envelope biogenesis” and “Nucleotide transport and metabolism”, and an impoverishment (less than 0.67 fold) in genes annotated into “Replication, recombination and repair” and “Cytoskeleton” (Supplementary data 3). Indeed, the Fisher's exact test revealed that both “Secondary metabolites biosynthesis, transport and catabolism” and “Amino acid transport and metabolism” functional classes were significantly enriched in positively selected genes, while the “Cytoskeleton” was significantly impoverished.

For the *basidioPAML_Hv* dataset, KOG annotation relative enrichment (over 1.5 folds) was found in genes as assigned to “Secondary metabolites biosynthesis, transport and catabolism”, “Cell cycle control, cell division, chromosome partitioning” and “Amino acid transport and metabolism”, as well as an impoverishment (less than 0.67 fold) in genes annotated as “Inorganic ion transport and metabolism”, “Transcription”, “RNA processing and modification”, “Intracellular trafficking, secretion, and vesicular transport”, “Signal transduction mechanisms”, “Cytoskeleton”, “Cell wall/membrane/envelope biogenesis”, “Nuclear structure” and “Chromatin structure and dynamics” (Fig 4). However, no functional classes were significantly over or under represented in this data set.

To allow a direct comparison between the *basidioPAML* and *basidioPAML_Hv* data sets, we performed the same enrichment analysis for the *basidioPAML* data set with the 531 non-saturated shared genes among data sets as reference. Similarly to the respective whole set of orthologous, genes annotated as “Secondary metabolites biosynthesis, transport and catabolism” and “Amino acid transport and metabolism” were enriched as well as “Coenzyme transport and metabolism”. Genes annotated as “Replication, recombination and repair” and “Cytoskeleton” were also found to be impoverished, in addition to “Intracellular trafficking, secretion and vesicular transport”. Fisher's exact test also reported a statistically significant enrichment and impoverishment of the same functional classes as the full data set.

Among the studied 985 genes, 249 blasted with genes involved in pathogenicity at PHI-base. Out of the 216 positively selected genes in the *basidioPAML*, 70 presented homology with entries in the PHI-base as well as 35 out of the 100 genes under positive selection in the *BasidioOnly_wHv*. In both data sets, the Fisher's exact test revealed that positively selected genes were enriched for PHI-base assigned genes putatively involved in pathogenicity, with p-values of 2.3e-6 and 4.0e-3 for *basidioPAML* and *basidioPAML_Hv*, respectively.
